# Supplementary material for: Identification of photoperiod-induced specific miRNAs in the adrenal glands of Sunite sheep (Ovis aries)
Source: Front Vet Sci. 2022 Jul 22;9:888207. doi: 10.3389/fvets.2022.888207 (PMC9354845; doi:10.3389/fvets.2022.888207)
Supplement: Supplementary file 1 [file Data_Sheet_1.ZIP › Supplementary file/miRDeep2 file.zip!/miRDeep2 file/Novel_Structure/SP42A1/Novel_40.pdf]

Provisional ID	:	NC_019460.2_5856
Score total	:	378.4
Score for star read(s)	:	3.9
Score for read counts	:	370.8
Score for mfe	:	2.1
Score for randfold	:	1.6
Score for cons. seed	:	
Total read count	:	739
Mature read count	:	555
Loop read count	:	0
Star read count	:	184

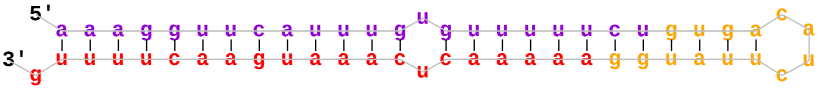

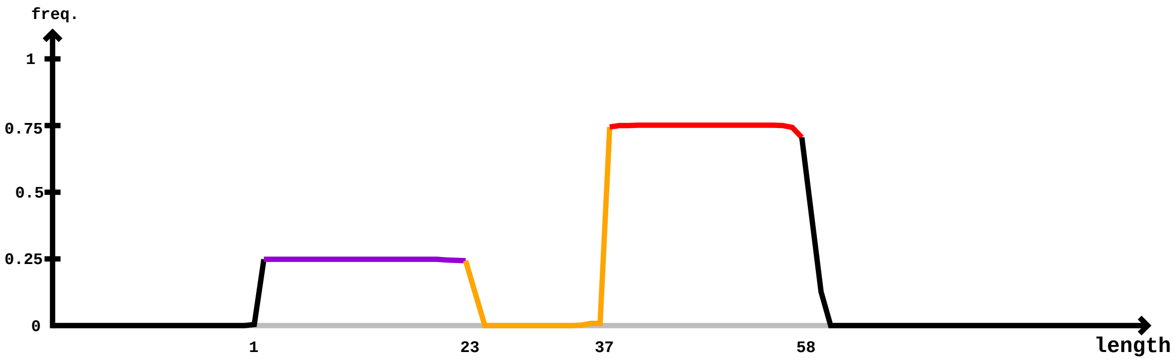

Star Mature

```
5'- aaauaauacuggguuggccaaaagguucauuuguguuuuucugugacaucuuauggaaaaacucaaaugaacuuuugggccaacccaaugaaaagcuccaaucccuucacuc -3'  obs
    aaauaauacuggguuggccaaaagguucauuuguguuuuucugugacaucuuauggaaaaacucaaaugaacuuuugggccaacccaaugaaaagcuccaaucccuucacuc       exp
    .........((((((((((.(((((((((((((.((((((((((((....)))))))))))).))))))))))))).)))))))))).........................  reads  mm  sample
    ...................aaaagguucauuugGuuuu..........................................................................  1    1  seq
    ...................aaaagguucauuuguguuuuu........................................................................  1    0  seq
    ...................aaaagguucauuuguguuuuuc.......................................................................  1    0  seq
    ....................aaagguucauuugGuuuu..........................................................................  1    1  seq
    ....................aaagguucauuuguguuuuucu......................................................................  180  0  seq
    .....................................................uggaaaaacCcaaaugaacuu......................................  1    1  seq
    ......................................................ggaaaaacCcaaaugaacuuu.....................................  5    1  seq
    ........................................................aaaaacCcaaaugaacuuuu....................................  2    1  seq
    ........................................................aaaaacucaaaugaacuuuu....................................  22   0  seq
    ........................................................aaaaacucaaaugaacuuuG....................................  2    1  seq
    ........................................................aaaaacucaaaugaacuuuC....................................  1    1  seq
    ........................................................Gaaaacucaaaugaacuuuug...................................  2    1  seq
    ........................................................aaaaacucaaaugaacuuuuU...................................  4    1  seq
    ........................................................aaaaacucaaaugaacCuuug...................................  2    1  seq
    ........................................................aaaaacucaaaugaacuuuug...................................  410  0  seq
    ........................................................aaaaacucaGaugaacuuuug...................................  2    1  seq
    ........................................................aaaaGcucaaaugaacuuuug...................................  3    1  seq
    ........................................................aGaaacucaaaugaacuuuugg..................................  2    1  seq
    ........................................................aaaaacucaaaugaacuuuuggU.................................  83   1  seq
    ........................................................aaaaacucaaaugaacuuuuggC.................................  10   1  seq
    .........................................................aaaacucaGaugaacuuuu....................................  1    1  seq
    .........................................................aaaacucaaaugaacuuuug...................................  1    0  seq
    .........................................................aaaacucGaaugaacuuuug...................................  1    1  seq
    .........................................................aaaacucaaaugaacuuuuggU.................................  1    1  seq
    ...........................................................aacucaaaugaacuuuug...................................  1    0  seq
```
